# Medical Decision Style and COVID-19 Behavior

**DOI:** 10.1177/0272989X221079354

**Published:** 2022-02-16

**Authors:** Gustav Tinghög, Liam Strand

**Affiliations:** Department of Management and Engineering, Division of Economics, Linköping University, Linköping, Sweden; National Center for Health Care Priority Setting, The Department of Health, Medicine and Caring Sciences (HMV), Linköping University, Linköping, Sweden; National Center for Health Care Priority Setting, The Department of Health, Medicine and Caring Sciences (HMV), Linköping University, Linköping, Sweden

**Keywords:** COVID-19, Medical Maximizer-Minimizer Scale, survey, social distancing, hygienic behavior

## Abstract

Given the flood of health-related information stirred up by the coronavirus disease 2019 (COVID-19) pandemic, it is important to understand the factors that influence people to engage in protective public health measures so that medical communication can be tailored to be effective. Following the idea that people have a general inclination toward health care utilization, which is either more passive (i.e., medical minimizer) or more aggressive (i.e., medical maximizer), we assess if this inclination extends to being more or less willing to engage in protective public health behavior. We investigate the effect of individual differences in medical minimizing and medical maximizing orientation on COVID-19–related protective behaviors and attitudes. We used the validated Medical Maximizer-Minimizer Scale (MMS) and surveyed a diverse opt-in sample of the Swedish population (*n* = 806). Our results show that the MMS significantly predicts a wide range of self-reported behaviors and attitudes in relation to COVID-19. Participants with a stronger minimization orientation were significantly less likely to practice social distancing, follow hygiene recommendations, and be supportive of strict COVID-19 policies. Participants with a stronger maximization orientation had a larger discrepancy between perceived own risk and others getting infected. Thus, they perceived themselves as being less at risk for getting infected compared to the average person. Our findings imply that the MMS can be effectively used to predict who is more or less reluctant to follow public health recommendations.

**JEL codes**: D70 E71 I12 I18

## Graphical abstract

**Graphical Abstract:**
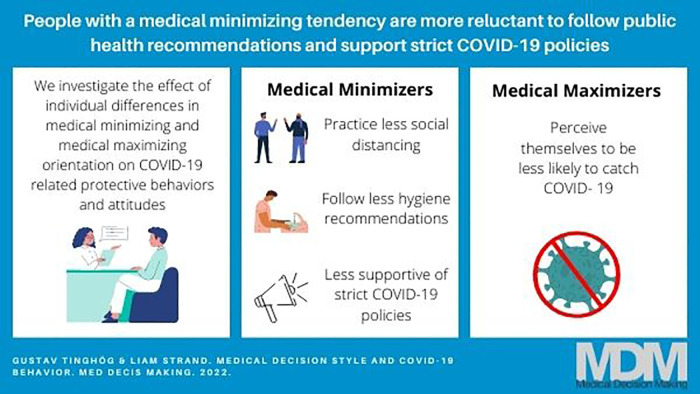
This is a visual representation of the abstract.

## Highlights

- We investigate the association between individual differences in medical minimizing and medical maximizing orientation and protective public health behaviors during coronavirus disease 2019 (COVID-19).
- The Medical Maximizer-Minimizer Scale can be effectively used to predict who is more or less reluctant to follow public health recommendations.
- Medical minimizers were less likely to practice social distancing, follow hygiene recommendations, and be supportive of strict COVID-19 policies.
- Medical maximizers perceived themselves as being less at risk of getting infected by COVID-19.

## Introduction

Following the coronavirus disease 2019 (COVID-19) outbreak, far-reaching disease prevention polices have been implemented worldwide. These include disease protective measures such as social distancing, frequent hand washing, wearing face masks, and lockdowns. The extent to which people follow these policies varies a lot between individuals. For example, medical conspiracy beliefs, relying on social media primarily as a source of information, and perceived risks of not getting infected by the virus have been linked to reluctance to engage in health-protective measures with regard to COVID-19.^1–3^ Moreover, inattention or failing to think sufficiently has been shown to make people less accurate when trying to identify misinformation related to COVID-19.^4^ Thus, there are strong reasons to believe that the way people approach and process information affects the level of protective measures people undertake during a pandemic such as the COVID-19 pandemic.

A possible contributing factor to why people process and respond differently to medical information is psychological differences in decision making style when it comes to health care utilization. Groopman and Hartzband^5^ characterized people as medical maximizers or medical minimizers. Medical maximizers are those who prefer a more aggressive approach to health care and proactively seek medical care also for minor ailments, while minimizers are those who prefer a more passive approach and avoid or delay seeking medical care until completely necessary. Based on this idea, Scherer et al.^6^ developed and validated the Medical Maximizer-Minimizer scale (MMS), which is a 10-item measurement scale designed to assess individual differences in general preferences toward wanting more v. less health care. The scale includes statements such as, “It is important to treat disease even when it does not make a difference in survival,” and “When it comes to medical treatment, more is usually better.” Distinct from distrust in medicine, the scale has been shown to predict general medical utilization and treatment preferences,^6^ demand for physician visits and medical tests,^7^ avoidance and delay of protective health care services,^8^ and preferences for both high- and low-benefit care.^9^ The general finding from this previous literature is that medical maximizers more frequently seek medical care in situations where costs are high and gain in terms of health outcome is low. It remains unclear, however, how the tendency to engage in medical maximizing is associated with risk perceptions and preventive behaviors during the COVID-19 pandemic.

As public health agencies want to make effective COVID-19 recommendations, they would want to know the effectiveness of these recommendation but also whether certain groups in society respond differently to these recommendations. Since the MMS has proven to predict general preferences for medical treatment, it seems plausible that it would also predict adherence to public health recommendations. Therefore, our objective is to test whether the MMS predicts COVID-19 behavior. More specifically, we test how medical maximizer-minimizer tendencies are associated with self-reported behavior and attitudes related to 1) social distancing, 2) hygienic behavior, 3) support of strict COVID-19 policies, and 4) the perceived risks of catching COVID-19.

## Method

We collected data through an online survey distributed to Swedish users registered at a voting advice application by the Swedish newspaper *Aftonbladet*, which is one of the most widely circulated newspapers in Sweden. (Data and analysis codes and a transcript of the survey instrument can be found on the project’s OSF repository at https://osf.io/mujdg/.) Participants in this subject pool voluntarily listed their e-mail addresses in 2010 for future surveys about political and social events.^10^ Thus, the subject pool is based on an opt-in sample and should not be taken as fully representative of the Swedish population. For this study, data were collected in 2 waves. The first wave was collected in May 2020 as part of an international collaboration on the social and moral psychology of COVID-19.^11^ The survey was sent out to the subject pool, which currently consists of 4,177 active subjects (34.4% females). The survey was closed for further participants once we reached our predetermined and prepaid sample size of 2,000 participants. Before the survey was closed, 18 additional participants responded, resulting in a sample of 2,018 participants. All outcome variables were collected in the first wave (i.e., social distancing, hygienic behavior, support of strict COVID-19 policies, and optimistic COVID-19 beliefs). The second wave was conducted during late August early September 2020, when we collected our main independent variable (i.e., the MMS). The questionnaire distributed in the second data wave was sent out only to the individuals who participated in the first data wave, so that responses could be linked at the individual level. In total, 806 (mean age: 57.6, females: 33.2%) participants responded to the questionnaire in the second data wave (40% response rate). Compared to the general population of Sweden, our sample was slightly older, had a higher share of males, and was more educated. Sample characteristics are shown in Supplemental Table S1.

Our 4 outcome measures were all part of the international collaboration on the social and moral psychology of COVID-19.^11^ To measure adherence to social distancing during the pandemic, we used 5 items (phrased as statements) to which respondents could agree or disagree. One such statement was as follows: “During the days of the coronavirus (COVID-19) pandemic, I have been staying at home as much as practically possible.” Five items were used to assess adherence to physical hygiene recommendations; a statement was, “During the days of the coronavirus (COVID-19) pandemic, I have been washing my hands longer than usual.” Five items were used to assess attitudes toward strict COVID-19 policies; a statement was, “During the days of the coronavirus (COVID-19) pandemic, I have been in favor of closing all schools and universities.” Participants responded on a slider between 0 (*strongly disagree*) and 10 (*strongly agree*), where the slider started at 5 (*neither agree nor disagree*). To assess perceived risk of catching COVID-19, we asked, “By the April 30th, 2021: How probable do you think it is that *you* have been infected by the coronavirus (COVID-19)?” We substituted *you* with *the average person in Sweden* when asking of the average perceived risk. The subject answered on a slider scale ranging from 0 (*no risk*) to 100 (*certain*), starting at 50. A variable for *optimistic/pessimistic beliefs* was calculated by subtracting an individual’s perceived own risk to catch COVID-19 with the perceived risk that the average person catches COVID-19. All items and are reported in the supplemental materials.

We used the MMS,^6^ translated to Swedish, to measure to what extent our subjects were medical maximizers or minimizers (Table 1). The respondents answered on a Likert 7-point scale on how much they agreed with the statements presented in Table 1 (1 = *strongly disagree*, 7 = *strongly agree*). The overall MMS value for each participant was calculated by averaging all item scores. This MMS score was used as a continuous variable in the analyses. A greater score indicates a stronger orientation toward being a medical maximizer, and a lower score indicates a tendency toward being a medical minimizer. To verify that participants were reading the instructions, we included an attention check where participants were asked to use a slider (which ranged from 0 to 100 and was initially set at 50) and indicate the value 0. All scales used are provided in the supplemental materials.

**Table 1:** The Medical Maximizer-Minimizer Scale (MMS)^a^

Item	Mean Score	SD
1. It is important to treat a disease even when it does not make a difference in survival.	4.19	1.70
2. It is important to treat a disease even when it does not make a difference in quality of life.	3.96	1.68
3. Doing everything to fight illness is always the right choice.	4.67	1.76
4. When it comes to health care, the only responsible thing to do is to actively seek medical care.	4.23	1.62
5. If I have a health issue, my preference is to wait and see if the problem gets better on its own.^b^	2.47	1.30
6. If I feel unhealthy, the first thing that I do is to go to the doctor and get a prescription.	2.08	1.27
7. I often suggest that friends and family see their doctor.	3.87	1.62
8. When it comes to health care, watching and waiting is never an acceptable option.	3.75	1.73
9. If I have a medical problem, my preference is to go straight to a doctor and ask for his or her opinion.	4.10	1.86
10. When it comes to medical treatment, more is usually better.	3.26	1.43
Medical Maximizer-Minimizer score average	3.66	0.97

a Respondents answered on a Likert 7-point scale on how much they agreed with each statement (1 = *strongly disagree*, 7 = *strongly agree*). A greater score indicates a stronger orientation toward being a medical maximizer, and a lower score indicates a tendency toward being a medical minimizer.

b Item was reverse coded.

Data were analyzed using a linear regression with robust standard errors (outlined in equation (1)) for each of our 4 dependent variables. To control for other factors, we thereafter regressed equation (1) but included $B_{j}X_{i}$ , which is a vector of our control variables age, gender, and education. Finally, we controlled the robustness of our results by controlling for subjects who had failed the attention check by adding a dichotomous variable, which equaled 1 if the participant had succeeded the attention check and 0 otherwise. Analysis was conducted using R.

(1) $$\mathrm{Dependent}\;{\mathrm{variable}}_{i}=\beta_{0}+\beta_{1}{\mathrm{MMS}}_{i}+\varepsilon_{i}$$

## Results

**Figure 1:**
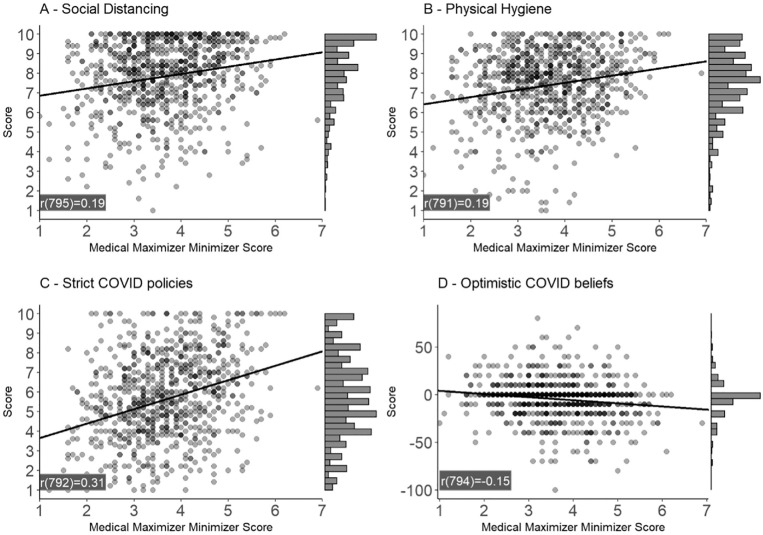
The correlation between Medical Maximizer-Minimizer Scale (MMS) score self-reported coronavirus disease 2019 (COVID-19) behavior and attitudes. A higher MMS score (maximum 7) indicates a stronger orientation toward being a medical maximizer while a lower score (minimum 1) indicates a stronger orientation toward being a medical minimizer. The histograms on the right side of each graph depict the distribution of responses for each outcome variable. Values presented in graphs indicate Pearson’s correlation coefficient (all significant at *P* < 0.001).

Figure 1 shows the unadjusted correlation between the MMS score and our 4 outcome variables: 1) social distancing, 2) hygienic behavior, 3) support of strict COVID-19 policies, and 4) optimistic COVID-19 beliefs. As shown in the figure, stronger medical maximizing tendencies were significantly associated with adherence to protective public health measures during the COVID-19 pandemic. It was also significantly associated with more optimistic beliefs about the risk of oneself getting infected compared to others and support for strict COVID-19 policies. Next, we present results from our regression model for each outcome variable adjusting for age, gender, and level of education.

### Social Distancing and Hygienic Behavior

Table 2 shows the results for social distancing and hygienic behavior as protective measures against COVID-19. The MMS score was significantly associated with people’s reluctance to practice social distancing. An additional score on the MMS (i.e., an increased tendency toward medical maximizing) was associated with an increase of 0.37 points on social distancing (*P* < 0.001). Table 2 also shows how the MMS score significantly predicted the extent to which people undertake precautionary hygienic measures to prevent the spread of the virus. A 1-point higher score on the MMS was associated with an 0.37-point increase on adherence to physical hygiene recommendations (*P* < 0.001). In addition, the results suggest that males were less likely to engage in protective measures during the COVID-19 pandemic and that younger participants (age 18+) were less likely to practice social distancing. All results held even after excluding participants who failed the attention check (see Suppl. Tables S2 and S3).

**Table 2:** Adherence to Protective Measures during the COVID-19 Pandemic^a^

Characteristic	Social Distancing			Physical Hygiene		
	Model 1Coefficient (SE)	Model 2Coefficient (SE)	StandardizedCoefficient (SE)	Model 1Coefficient (SE)	Model 2Coefficient (SE)	StandardizedCoefficient (SE)
MMS score	0.37 (0.07)***	0.30 (0.08)***	0.16	0.37 (0.07)***	0.42 (0.07)***	0.22
Age		0.02 (0.005)***	0.14		−0.01 (0.01)	−0.05
Male		−0.71 (0.13)***	−0.18		−0.94 (0.13)***	−0.24
At most primary school		0.11 (0.29)	0.02		−0.08 (0.31)	−0.01
Secondary school		−0.37 (0.17)*	−0.08		−0.35 (0.17)*	−0.08
Higher education (<2 years)		0.12 (0.15)	0.03		0.21 (0.17)	0.05
Constant	6.48 (0.28)***	6.19 (0.34)***		6.05 (0.27)***	6.89 (0.35)***	
Observations	797	761	761	793	761	761
*R* ^2^	0.04	0.1		0.04	0.11	

a All regressions are ordinary least squares with robust standard errors. Social distancing was measured on a scale of 0 to 10 (maximal adherence). Physical hygiene was measured on a scale between 0 and 10 (maximal adherence). Medical Maximizer-Minimizer Scale (MMS) score was measured on a scale of 1 to 7. A higher MMS score indicates a stronger orientation toward being a medical maximizer while a lower score indicates a stronger orientation toward being a medical minimizer. Higher education (>3 years) is the reference group for education.

* *P* < 0.05. **P < 0.01. ***P < 0.001.

### Support of Strict COVID-19 Policies and the Perceived Risks of Catching COVID-19

Table 3 shows the results for attitudes toward strict public health policies and perceived risks of catching COVID-19. Participants with a tendency toward maximization orientation were significantly more likely to support stricter COVID-19 policies (such as lockdowns of schools and universities and bans on nonnecessary travels). A 1-point increase on the MMS (i.e., an increased tendency toward medical maximizing) was correlated with a 0.74-point increase on the support of strict COVID-19 policies scale. Table 3 also shows how the MMS score was associated with beliefs about the risk of catching COVID-19. In general, the perceived likelihood of getting infected was 4.62 percentage points lower when assessing the risk for oneself compared to when assessing the risk for an average adult of the Swedish population (mean = −4.62, *t* = −6.36, *P* < 0.001), indicating a more optimistic belief about the risk of oneself getting infected compared to others. A greater orientation toward medical maximizing was also associated with more optimistic beliefs about the possibility of getting infected—a 1-point increase on the MMS resulted in a 3.29 percentage point greater perceived difference (*P* < 0.001). All results were robust for excluding participants who failed the attention check.

**Table 3:** Support of Strict COVID-19 Policies and Perceived Risk of Catching the Virus during the COVID-19 Pandemic^a^

Characteristic	Support of Strict COVID-19 Policies			Optimistic COVID-19 Belief		
	Model 1Coefficient (SE)	Model 2Coefficient (SE)	StandardizedCoefficient (SE)	Model 1Coefficient (SE)	Model 2Coefficient (SE)	StandardizedCoefficient (SE)
MMS score	0.74 (0.09)***	0.74 (0.09)***	0.31	−3.29 (0.72)***	−2.72 (0.76)***	−0.13
Age		−0.01 (0.01)*	−0.08		−0.20 (0.05)***	−0.14
Male		−0.16 (0.17)	−0.03		3.20 (1.59)*	0.07
At most primary school		0.76 (0.35)*	0.09		1.37 (3.06)	0.02
Secondary school		0.35 (0.22)	0.06		−0.24 (1.78)	−0.005
Higher education (<2 years)		0.84 (0.20)***	0.16		−2.34 (1.87)	−0.05
Constant	2.92 (0.32)***	3.42 (0.40)***		7.40 (2.71)**	14.69 (3.76)***	
Observations	794	760	760	796	760	760
*R* ^2^	0.10	0.13		0.02	0.06	

a All regressions are ordinary least squares with robust standard errors. Support of strict coronavirus disease 2019 (COVID-19) policies was measured on a scale of 0 to 10 (maximal support for strict COVID-19 policies). Optimistic COVID-19 belief was calculated as the perceived probability that the subject would catch COVID-19 subtracted by the perceived probability that the average person would catch COVID-19. Thus, a negative value indicates a more optimistic belief while a positive value indicates a more pessimistic belief. Medical Maximizer-Minimizer Scale (MMS) score was measured on a scale of 1 to 7. A higher MMS score indicates a stronger orientation toward being a medical maximizer while a lower score indicates a stronger orientation toward being a medical minimizer. Higher education (>3 years) is the reference group for education.

* *P* < 0.05. **P < 0.01. ***P < 0.001.

## Discussion

Our results show that the MMS predicts who is more or less reluctant to engage in protective public health measures in the case of a worldwide pandemic such as the COVID-19. People with a tendency toward approaching medical utilization as minimizers (i.e., those who have a tendency to seek health care only when completely necessary) were less likely engage in health-protective measures. Previous work has shown that people who have a tendency toward medical minimization have a lower willingness to seek out medical care in situations where costs are high and gain in terms of health outcome is low.^6–9^ We expand on this research by showing that the MMS predicts who will follow public health recommendations issued in response to a global public health crisis—a situation where the cost of health-promoting behaviors in terms of effort is low and the possible gain in terms of health outcome is high. We also find that medical maximizers are more likely to support strict public health policies and believe that they are less likely to catch COVID-19 compared to medical minimizers. In a recent study, van Bavel et al.^11^ found that people who identified more strongly with their nation consistently reported engagement in public health behaviors and greater support for public health policies. Our study extends these results by showing that general inclination toward health care utilization can also help to explain when and why people are reluctant to engage in health-protective measures and support for strict public health policies.

Our results have practical implications for fighting the spread of COVID-19 now and similar viruses in the future. Insights about who is more and less reluctant to follow public health recommendations is an important first step for making public health messaging more effective. Theoretically, our findings shed further light on the importance of decision style and how people process medical information. Following the idea that decision making is best described as an adaptive process where people constantly make tradeoffs between risk for bad outcomes and effort,^12^ it is possible that medical minimizers process information in an intuitive and less exhaustive way, thus putting more weight on effort rather than risk minimizing in their everyday decision making. In the context of COVID-19, this could imply an increased tendency to be distracted and inattentive, which may explain why minimizers are less likely to follow public health recommendations. When faced with a decision situation, it could potentially be easier for minimizers to ignore recommendations such as social distancing because other issues are less abstract and more attention grabbing at the point of decision. However, the opposite interpretation is also plausible (i.e., that medical maximizers process information in a less exhaustive way than minimizers). Medical maximizers might be more inclined to take action to reduce risk without thinking much about the existing evidence, the likelihood of benefit, or the inconvenience of the intervention. By contrast, minimizers may therefore be more likely to actively think about these tradeoffs.

When our findings are considered together with other recent findings showing that medical maximizers are more susceptible to misinformation regarding COVID-19^4^ and health in general,^13^ a complex pattern of results emerges. This complex pattern raises some interesting questions for future research. In particular, how can we understand the puzzling fact that medical maximizers are more willing to engage in protective health measures but also more like to believe misinformation? We can only speculate about this, but an interpretation consistent with existing empirical findings is that medical maximizers are more susceptible to medical information in general—both good and bad. Thus, medical maximizers possibly follow a “more is always better” heuristic, while medical minimizers are more likely to engage in willful ignorance when processing medical information. The arising complex pattern also reinforces our general belief that no single decision style or decision mode is unequivocally good or bad when it comes to medical decision making. Instead, it is the adaptive ability to switch between different decision styles and modes depending on the situation that is likely to be key for good medical decision making.

There are a few limitations with the study. The first is that all results are correlational in nature, and we therefore cannot establish causal directions of the effects that we find. We also rely on self-reported measures. Thus, we do not know to what extent participants answer truthfully. It could be that participants, in general, report that they behave according to recommendations to a higher degree than they actually do. However, there is little reason to believe that maximizers and minimizers should differ with regard to how truthful they are when responding. Another potential limitation is that data were collected in 2 waves (data on behavior during the pandemic were collected in the first wave and data on individual differences in medical decision style in the second wave). Although the MMS aims to measure a stable trait or decision style, studies have shown that personality characteristics may change in response to dramatic events such as becoming unemployed.^14^ Thus, we cannot rule out the possibility that participants changed their decision style during the 3 months between wave 1 and wave 2. Having separated data collections for our dependent and independent variables can, however, also be seen as strength since this limits the possibility that participants knew the underlying research question and adjusted their responses accordingly. Finally, since we used an opt-in survey to measure attitudes toward protective public health measures, these results may not be fully indicative for the general Swedish population.

To sum up, we found evidence that the MMS can be used to predict self-reported COVID-19 behaviors. Medical maximizers were more likely to practice social distancing, follow hygiene recommendation, and support strict COVID-19 policies. Medical minimizers were more reluctant to follow public health recommendations. As the time horizon of the pandemic is yet unknown, policy makers should make use of these results to tailor messaging and target groups with specific recommendations.

## Supplemental Material

sj-docx-1-mdm-10.1177_0272989X221079354 – Supplemental material for Medical Decision Style and COVID-19 Behavior Click here for additional data file. Supplemental material, sj-docx-1-mdm-10.1177_0272989X221079354 for Medical Decision Style and COVID-19 Behavior by Gustav Tinghög and Liam Strand in Medical Decision Making
