# Supplementary material for: Medical Decision Style and COVID-19 Behavior
Source: Med Decis Making. 2022 Feb 16;42(6):776–82. doi: 10.1177/0272989X221079354 (PMC9326342; doi:10.1177/0272989X221079354)
Supplement: sj-docx-1-mdm-10.1177_0272989X221079354 – Supplemental material for Medical Decision Style and COVID-19 Behavior [file sj-docx-1-mdm-10.1177_0272989X221079354.docx]

**Supplementary material**

Medical Decision Style and COVID-19 Behavior –

Gustav Tinghög and Liam Strand

*S1. Additional analyzes referenced in the paper*

*S2. Transcript of scales used in the paper*

S1. Additional analyzes referenced in the results section

Medical Maximizer Minimizer predicts COVID-19 behavior

Table S1 – Sample characteristics

|  | Wave 1 | Wave 2 | test for difference between wave 1 and wave 2 | Sweden |
| --- | --- | --- | --- | --- |
| n | 2018 | 806 |  | N/A |
| Age, mean (SD) | 52.66 (15.72) | 57.56 (14.23) | t(1678)=-7.62, p<.001 | 50.49 (N/A)^a^ |
| MISSING, % (n) | 19.8% (399) | 3.5% (28) |  |  |
| Gender |  |  | X^2^(1, N=2411)=15.3, p<.001 |  |
| Male, % (n) | 47.1% (951) | 65.3% (526) |  | 50.3%^b^ |
| Female, % (n) | 33.3% (673) | 32.4% (261) |  |  |
| MISSING, % (n) | 19.5% (394) | 2.4% (19) |  |  |
| Education |  |  | X^2^(4, N=2403)=1.46, p=.692 |  |
| At most primary school, % (n) | 5.6% (112) | 7.3% (59) |  | 11.3% (580 290)^c^ |
| Secondary school, % (n) | 16.2% (326) | 19.0% (153) |  | 43.9% (2 255 050)^c^ |
| Higher education (<2 years), % (n) | 17.6% (356) | 23.0% (185) |  | 16.0% (820 151)^c^ |
| Higher education (>3 years), % (n) | 41.0% (828) | 47.6% (384) |  | 28.9% (1 487 106)^c^ |
| MISSING, % (n) | 19.6% (396) | 3.1% (25) |  |  |
| Social Distancing, mean (SD) | 7.74 (1.82) | 7.84 (1.84) | t(1544)=-1.17, p=.242 |  |
| MISSING, % (n) | 15.8% (318) | 1.1% (9) |  |  |
| Physical Hygiene, mean (SD) | 7.52 (1.81) | 7.38 (1.85) | t(1513)=1.76, p=.079 |  |
| MISSING, n (%) | 14.6% (295) | 1.6% (13) |  |  |
| Strict COVID Policies, mean (SD) | 5.68 (2.25) | 5.62 (2.30) | t(1509)=0.68, p=.494 |  |
| MISSING, n (%) | 14.0% (283) | 1.5% (12) |  |  |
| Optimistic COVID beliefs, mean (SD) | -4.46 (21.26) | -4.62 (20.51) | t(1596)=0.18, p=.857 |  |
| MISSING, n (%) | 14.3 (288) | 1.2% (10) |  |  |

Note: ^a^SCB (2020b) ^b^SCB (2020a). ^c^SCB (2020c). Social distancing was measured on a scale of 0 to 10 (maximal adherence). Physical hygiene was measured on a scale between 0-10 (maximal adherence). MMS (Medical Maximizer-Minimizer Scale) was measured on a scale of 1 to 7. Support of strict COVID-19 policies was measured on a scale of 0 to 10 (maximal support for strict COVID-19 policies). Optimistic belief was calculated as the perceived probability that the subject would catch COVID-19 subtracted by the perceived probability that the average person would catch COVID-19. Thus, a negative value indicates more optimistic beliefs while a positive value indicates more pessimistic beliefs. A higher MMS score indicates a stronger orientation toward being a medical maximizer while a lower score indicates a stronger orientation toward being a medical minimizer.

Table S2 – Adherence to protective measures during the COVID-19 pandemic. Excluding those who fail attention check.

|  | Social Distancing | | | Physical Hygiene | | |
| --- | --- | --- | --- | --- | --- | --- |
|  | Model 1 | Model 2 | Standardized | Model 1 | Model 2 | Standardized |
| MMS | 0.40^***^ (0.08) | 0.32^***^ (0.08) | 0.17 | 0.35^***^ (0.07) | 0.40^***^ (0.08) | 0.20 |
| Age |  | 0.02^***^ (0.005) | 0.13 |  | -0.01 (0.01) | -0.06 |
| Male |  | -0.69^***^ (0.13) | -0.18 |  | -0.91^***^ (0.14) | -0.23 |
| At most primary school |  | 0.13 (0.31) | 0.02 |  | -0.06 (0.33) | -0.01 |
| Secondary school |  | -0.25 (0.18) | -0.05 |  | -0.29 (0.19) | -0.06 |
| Higher education (<2 years) |  | 0.09 (0.16) | 0.02 |  | 0.14 (0.18) | 0.03 |
| Constant | 6.46^***^ (0.29) | 6.23^***^ (0.36) |  | 6.09^***^ (0.29) | 6.99^***^ (0.38) |  |
| Observations | 711 | 685 | 685 | 710 | 685 | 685 |
| R^2^ | 0.04 | 0.10 |  | 0.03 | 0.09 |  |

Note: All regressions are ordinary least square with robust standard errors. Social distancing was measured on a scale of 0 to 10 (maximal adherence). Physical hygiene was measured on a scale between 0-10 (maximal adherence). MMS (Medical Maximizing-Minimizing Scale) was measured on a scale of 1 to 7. A higher MMS score indicates a stronger orientation toward being a medical maximizer while a lower score indicates a stronger orientation toward being a medical minimizer. Higher education (>3 years) is the reference group for education. *** p < 0.001, ** p < 0.01. * p < 0.05.

Table S3 – Support of strict COVID-19 policies and perceived risk of catching the virus during the COVID-19 pandemic. Excluding those who fail attention check.

|  | Strict COVID policies | | | Optimistic COVID beliefs | | |
| --- | --- | --- | --- | --- | --- | --- |
|  | Model 1 | Model 2 | Standardized | Model 1 | Model 2 | Standardized |
| MMS | 0.73^***^ (0.09) | 0.74^***^ (0.10) | 0.30 | -2.87^***^ (0.77) | -2.12^**^ (0.81) | -0.10 |
| Age |  | -0.01* (0.01) | -0.08 |  | -0.21^***^ (0.05) | -0.15 |
| Male |  | -0.07 (0.17) | -0.01 |  | 3.35^*^ (1.67) | 0.08 |
| At most primary school |  | 0.60 (0.37) | 0.07 |  | 3.23 (3.01) | 0.04 |
| Secondary school |  | 0.37 (0.23) | 0.06 |  | -0.29 (1.85) | -0.01 |
| Higher education (<2 years) |  | 0.84^***^ (0.21) | 0.16 |  | -2.61 (1.98) | -0.05 |
| Constant | 2.92^***^ (0.34) | 3.35^***^ (0.44) |  | 6.36^*^ (2.87) | 13.59^***^ (3.77) |  |
| Observations | 709 | 684 | 684 | 710 | 684 | 684 |
| R^2^ | 0.09 | 0.12 |  | 0.02 | 0.05 |  |

Note: All regressions are ordinary least square with robust standard errors. Support of strict COVID-19 policies was measured on a scale of 0 to 10 (maximal support for strict COVID-19 policies). Optimistic beliefs was calculated as the perceived probability that the subject would catch COVID-19 subtracted by the perceived probability that the average person would catch COVID-19. Thus, a negative value indicates more optimistic beliefs while a positive value indicates more pessimistic beliefs. MMS (Medical Maximizing-Minimizing scale) was measured on a scale of 1 to 7. A higher MMS score indicates a stronger orientation toward being a medical maximizer while a lower score indicates a stronger orientation toward being a medical minimizer. Higher education (>3 years) is the reference group for education. *** p < 0.001, ** p < 0.01. * p < 0.05.

S2. Transcript of scales

[Medical Maximizer-Minimizer Scale]

Please indicate how much you agree or disagree with the statements below. There are no right or wrong answers, only respond in such a way that reflects your own personal opinions.

|  | Strongly disagree | Disagree | Somewhat disagree | Neither agree nor disagree | Somewhat agree | Agree | Strongly agree |
| --- | --- | --- | --- | --- | --- | --- | --- |
| It is important to treat a disease even when it does not make a difference in survival |  |  |  |  |  |  |  |
| It is important to treat a disease even when it does not make a difference in quality of life |  |  |  |  |  |  |  |
| Doing everything to fight illness is always the right choice |  |  |  |  |  |  |  |
| When it comes to health care, the only responsible thing to do is to actively seek medical care |  |  |  |  |  |  |  |
| If I have a health issue, my preference is to wait and see if the problem gets better on its own |  |  |  |  |  |  |  |
| If I feel unhealthy, the first thing that I do is to go to the doctor and get a prescription |  |  |  |  |  |  |  |
| I often suggest that friends and family see their doctor |  |  |  |  |  |  |  |
| When it comes to health care, watching and waiting is never an acceptable option |  |  |  |  |  |  |  |
| IF I have a medical problem, my preference is to go straight to a doctor and ask for his or her opinion |  |  |  |  |  |  |  |
| When it comes to medical treatment, more is usually better |  |  |  |  |  |  |  |

[Social distancing]

**Be as precise as you can:**During the days of the coronavirus (COVID-19) pandemic, I have…

|  | **Strongly disagree** | **Neither agree nor disagree** | **Strongly agree** |
| --- | --- | --- | --- |

|  | 0 | 1 | 2 | 3 | 4 | 5 | 6 | 7 | 8 | 9 | 10 |
| --- | --- | --- | --- | --- | --- | --- | --- | --- | --- | --- | --- |

| Been staying at home as much as practically possible () | 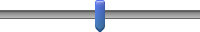 |
| --- | --- |
| Visited friends, family , or colleagues outside of my home () | 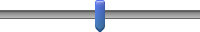 |
| Limited the number of visits to the grocery store to an absolute minimum () | 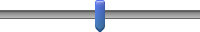 |
| Kept physical distance to all other people outside of my home () | 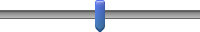 |
| Avoided to shake hands with people outside of my home () | 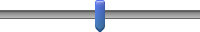 |

[Physical hygiene]

**Be as precise as you can:**During the days of the coronavirus (COVID-19) pandemic, I have…

|  | **Strongly disagree** | **Neither agree nor disagree** | **Strongly agree** |
| --- | --- | --- | --- |

|  | 0 | 1 | 2 | 3 | 4 | 5 | 6 | 7 | 8 | 9 | 10 |
| --- | --- | --- | --- | --- | --- | --- | --- | --- | --- | --- | --- |

| Been washing my hands longer than usual () | 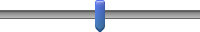 |
| --- | --- |
| Been washing my hands (with soap) more carefully than regular () | 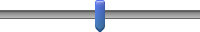 |
| Washed by hands immediately when I have returned home () | 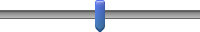 |
| Disinfected objects which are often used, such as mobile phones and keys () | 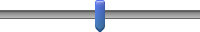 |
| Sneezed and coughed into the elbow () | 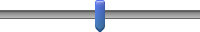 |

[Strict COVID policies]

**Be as precise as you can:** During the days of the coronavirus (COVID-19) pandemic, I have…

|  | **Strongly disagree** | **Neither agree nor disagree** | **Strongly agree** |
| --- | --- | --- | --- |

|  | 0 | 1 | 2 | 3 | 4 | 5 | 6 | 7 | 8 | 9 | 10 |
| --- | --- | --- | --- | --- | --- | --- | --- | --- | --- | --- | --- |

| Been in favor of closing all schools and universities () | 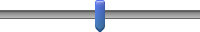 |
| --- | --- |
| Been in favor of closing all bars and restaurants () | 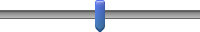 |
| Been in favor of closing all parks () | 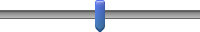 |
| Been in favor of prohibitions of public gatherings where a lot of people meet on a spot (sports and culture) () | 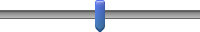 |
| Been in favor of prohibitions of all non-essential travels () | 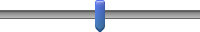 |

[Optimistic COVID beliefs]

Please respond to the following questions as precise as possible:

|  | **0% = Impossible** | **100% = Certain** |
| --- | --- | --- |

|  | 0 | 10 | 20 | 30 | 40 | 50 | 60 | 70 | 80 | 90 | 100 |
| --- | --- | --- | --- | --- | --- | --- | --- | --- | --- | --- | --- |

| By the April 30th, 2021: How probable do you think it is that you have been infected by the coronavirus (covid-19?) () | 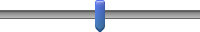 |
| --- | --- |
| By the April 30th, 2021: How probable do you think it is that the average person in Sweden has been infected by the coronavirus (covid-19?) () | 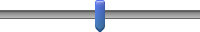 |

[Attention check]

Please confirm that you have read this question by moving the marker all the way to the left, so you give the value 0 as response

|  | **Strongly disagree** | **Neither agree nor disagree** | **Strongly agree** |
| --- | --- | --- | --- |

|  | 0 | 1 | 2 | 3 | 4 | 5 | 6 | 7 | 8 | 9 | 10 |
| --- | --- | --- | --- | --- | --- | --- | --- | --- | --- | --- | --- |

| Weather assessment () | 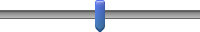 |
| --- | --- |

[Gender]

What is your gender?

- Male
- Female
- Other ________________________________________________

[Age]

How old are you?

________________________________________________________________

[Education]

What’s your highest completed education?

- Lack formal education
- *Volksschule*, 6 years
- *Realschule*, faculty school or girl school
- Primary school, 9 years
- Secondary school, matriculation examination
- Additional education after secondary school (e.g., qualified vocational training)
- Health school, social collage (older education)
- Bachelor’s degree or college degree/college engineer
- Master’s degree, civil engineer, or other similar longer education
- Postgraduate education (licentiate or PhD)

# References

SCB. (2021a). Sveriges befolkning. Available from: <https://www.scb.se/hitta-statistik/sverige-i-siffror/manniskorna-i-sverige/sveriges-befolkning/> [2021-05-04]

SCB. (2021b). Sveriges befolkningspyramid. Available from: <https://www.scb.se/hitta-statistik/sverige-i-siffror/manniskorna-i-sverige/sveriges-befolkningspyramid/> [2021-05-04]

SCB. (2021c). Utbildningsnivån i Sverige. Available from: <https://www.scb.se/hitta-statistik/sverige-i-siffror/utbildning-jobb-och-pengar/utbildningsnivan-i-sverige/> [2021-05-04]
